# Proteomics reveal a concerted upregulation of methionine metabolic pathway enzymes, and downregulation of carbonic anhydrase-III, in betaine supplemented ethanol-fed rats

**DOI:** 10.1016/j.bbrc.2009.02.082

**Published:** 2009-04-17

**Authors:** Kusum K. Kharbanda, Vasanthy Vigneswara, Benita L. McVicker, Anna U. Newlaczyl, Kevin Bailey, Dean Tuma, David E. Ray, Wayne G. Carter

**Affiliations:** a School of Biomedical Sciences, University of Nottingham, Queen’s Medical Centre, Nottingham NG7 2UH, UK; b Liver Study Unit, Department of Veterans Affairs Medical Centre, Omaha, NE 68105, USA; c Department of Internal Medicine, University of Nebraska Medical Centre, Omaha, NE 68198, USA

**Keywords:** Alcohol, Liver, Betaine, Proteomics, *S*-adenosylhomocysteine, *S*-adenosylmethionine, Carbonic anhydrase-III, Redox stress, Isoaspartate, Protein isoaspartyl methyltransferase

## Abstract

We employed a proteomic profiling strategy to examine the effects of ethanol and betaine diet supplementation on major liver protein level changes. Male Wistar rats were fed control, ethanol or betaine supplemented diets for 4 weeks. Livers were removed and liver cytosolic proteins resolved by one-dimensional and two-dimensional separation techniques. Significant upregulation of betaine homocysteine methyltransferase-1, methionine adenosyl transferase-1, and glycine *N*-methyltransferase were the most visually prominent protein changes observed in livers of rats fed the betaine supplemented ethanol diet. We hypothesise that this concerted upregulation of these methionine metabolic pathway enzymes is the protective mechanism by which betaine restores a normal metabolic ratio of liver *S*-adenosylmethionine to *S*-adenosylhomocysteine. Ethanol also induced significant downregulation of carbonic anhydrase-III protein levels which was not restored by betaine supplementation. Carbonic anhydrase-III can function to resist oxidative stress, and we therefore hypothesise that carbonic anhydrase-III protein levels compromised by ethanol consumption, contribute to ethanol-induced redox stress.

## Introduction

Alcoholic liver disease (ALD) is a major healthcare problem with worldwide prevalence. Our research group and others have modelled ALD by feeding animals a proportion of calories as ethanol resulting in the reproduction of many of the clinical manifestations seen in ALD; including the ethanol-induced disruption of liver enzyme activities and metabolite production associated with the methionine metabolic pathway [1–9], depletion of the major endogenous antioxidant glutathione, production of reactive oxygen species and aldehydic byproduct damage [10–15], and apoptosis [4,10,11,14,16].

Previous research has established that prolonged ethanol feeding affects both pathways that catalyse the remethylation of homocysteine to form methionine in the liver methionine metabolic pathway [1,5]; resulting in a lowering of hepatocyte *S*-adenosylmethionine (SAM) levels [1,7]. Additionally, ethanol-induced impaired homocysteine remethylation drives the thermodynamically favoured conversion of homocysteine (with adenosine) to *S*-adenosylhomocysteine (SAH); resulting in an elevation of hepatocyte SAH levels [6,7]. The consequential alteration in the hepatocellular SAM to SAH ratio can compromise activities of many methyltransferases, including protein isoaspartyl methyltransferase (PIMT), to result in an accumulation of damaged proteins bearing isoaspartate residues [9,17–20].

We and other research groups have shown that betaine diet supplementation has therapeutic potential to abrogate some of the ill effects of ethanol consumption [3,6,8,9,11,12,15]; including an ability to restore a normal SAM:SAH ratio [3,6,8,9] to protect the liver from ethanol-induced fatty infiltration [3,8], and prevent accumulation of isoaspartate damage in cytosolic proteins [9]. In order to further examine the influences of ethanol and betaine on other components of the methionine metabolic pathway and their molecular interactions, we adopted a proteomic approach to identify gross cytosolic liver protein changes in livers of rats fed control or ethanol diets in the presence and absence of betaine supplementation.

## Materials and methods

*Animal treatment.* Animals were fed Lieber DeCarli control and ethanol liquid diets with or without 1% betaine supplementation for 4 weeks as previously described [8]. At the time of sacrifice the liver was removed and immediately used for preparing subcellular fractions [8]. The care, use, and procedures performed on these rats were approved by the Institutional Animal Care and Use Committee at the Omaha Veterans Affairs Medical Center, USA.

*Protein concentrations.* Protein concentrations of liver homogenates were measured using the DC protein assay (Bio-Rad) using bovine serum albumin as a protein standard.

*One dimensional polyacrylamide gel electrophoresis (1D PAGE).* Liver homogenate proteins (20 μg/gel lane) were separated on 4–12% Bis–Tris NuPAGE Novex pre-cast gels run with MES running buffer as described previously [20]. Resolved proteins were either stained with Coomassie and photographed using a Fugi E900 digital camera followed by quantification using a GS-710 imaging densitometer (Bio-Rad) utilising Quantity One densitometric quantitation software (Bio-Rad), or transferred at 80 V for 2 h to a polyvinylidene difluoride (PVDF) membrane for Western blotting.

*Carbonic anhydrase-III (CA-III) Western blotting.* PVDF membranes were washed in a buffer of 20 mM K-MES, pH 6.2 containing 150 mM NaCl and 0.05% (v/v) Tween 20, blocked for 1 h at room temperature with 5% (w/v) milk fat in wash buffer, and then incubated overnight at 4 °C with a goat polyclonal CA-III primary antibody (Santa-Cruz, sc-50715) at a 1:250 dilution in wash buffer. Following blot washing and incubation with a secondary antibody (polyclonal rabbit anti-goat immunoglobulins-horseradish peroxide-conjugated (Dako, P0449) at 1:2000 dilution in wash buffer, CA-III localisation was visualised using SuperSignal West Pico Chemiluminescent substrate (Pierce) with the light generated captured on CL-Xposure X-ray film (Pierce). The levels of CA-III were quantified using a GS-70 densitometer and Quantity One software.

*One dimensional isoelectric focussing (1D IEF).* Cytosolic liver proteins (20 μg/gel lane) and IEF standards (Serva IEF markers, Invitrogen) were loaded onto vertical Novex, pH 3–10, IEF gels, and proteins resolved at constant voltage, initially for 1 h at 100 V, and then 1.5 h at 200 V. Proteins were fixed by washing gels in 12% (w/v) trichloroacetic acid, stained with Coomassie, and then photographed.

*Two dimensional polyacrylamide gel electrophoresis (2D-PAGE) and mass spectrometry.* For each analysis, six hundred μg of cytosolic liver protein was resolved by 2D-PAGE and separated proteins Coomassie stained and then photographed. Protein bands or spots from either, 1D PAGE, 1D IEF, or 2D-PAGE gels were excised and identified by matrix assisted laser-desorption ionisation-time of flight (MALDI-TOF) mass spectrometry or liquid chromatography mass spectrometry/mass spectrometry (LC-MS/MS). Procedures were similar to those described in a previous publication [20] and have been included as Supplementary Material.

## Results

### Analysis of liver protein levels following ethanol consumption and betaine supplementation

**Fig. 1:**
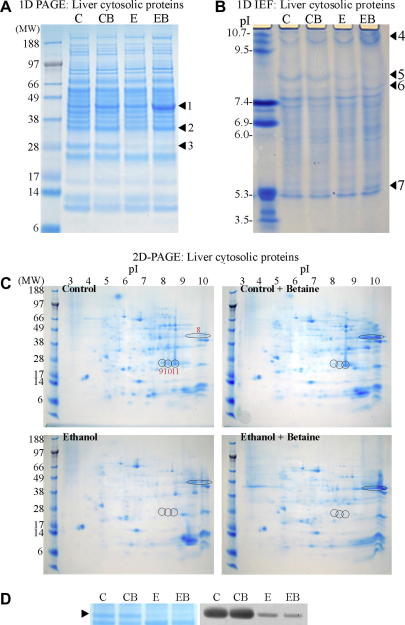
Proteomic profiling of liver cytosolic proteins. (A) Liver cytosolic proteins from rats fed a control (C), control with 1% betaine (CB), ethanol (E), or ethanol with 1% betaine (EB) diet were resolved by 1D PAGE, or (B) cytosolic liver proteins were resolved by vertical 1D IEF. Major protein level changes arising from the different feeding regimens are marked with arrowheads and labelled 1–3 for 1D PAGE and 4–7 for 1D IEF. (C) Liver cytosolic proteins from rats fed these four feeding diets were resolved by 2D-PAGE. Major protein level changes arising from the different feeding regimens are ringed and numbered 8–11 on the control diet gel. (D) Cytosolic liver proteins resolved by 1D PAGE were transferred to a PVDF membrane and Western blotted for CA-III protein levels. Ethanol-induced CA-III protein depletion evident from Coomassie protein staining (left panel) was supported by anti-CA-III Western blotting (right panel). Eight feeding sets of rats were used for protein separation by 1D PAGE, 1D IEF, or 2D-PAGE, with each individual rat sample resolved 1 or 2 times for IEF gels and 2–3 times for 1D- or 2D-PAGE. Protein staining patterns shown were reproducible with all samples. Western blotting for CA-III was performed on 5 sets of animals for liver cytosolic proteins.

In order to characterise the most prominent protein changes that arise in livers from rats fed control or ethanol-containing diets with or without betaine supplementation, cytosolic liver proteins were resolved by 1D PAGE. Three major protein level changes were apparent at denatured molecular weights of ∼45, ∼35, and ∼28 kDa (Fig. 1A, marked with arrowheads labelled 1–3 chronologically by decreasing molecular weight). Protein 1 levels were increased in the livers of ethanol-fed and betaine supplemented rats in comparison to the livers of control rats. This increase was highest (∼4-fold over controls) in the livers of the betaine supplemented ethanol-fed group. Protein 2 was also significantly upregulated (∼2-fold) in livers of both of the betaine supplemented diet groups. However, a downregulation of Protein 3 was observed in the livers of rats fed the ethanol diet and these levels were not restored by betaine supplementation.

To further characterise protein level changes, liver cytosolic proteins from these diet groups were also resolved by 1D IEF. Four clear gross protein level changes were evident (Fig. 1B, marked with arrowheads and labelled 4–7 in chronological order of their decreasing isoelectric points). Protein 4 had a pI of ∼10 with highest levels revealed in the livers of ethanol-fed and betaine supplemented animals. Protein 5 (pI ∼8.3) was downregulated in livers of both ethanol and betaine supplemented ethanol-fed rats. Betaine supplementation to the ethanol-fed rats resulted in upregulation of proteins labelled 6 and 7 of pIs of ∼8.0 and ∼5.5, respectively.

Cytosolic liver proteins from each of the four diet groups were also resolved by 2D-PAGE. The most prominent protein changes are ringed on each of the Coomassie stained gels from each diet group (Fig. 1C and labelled 8–11 on the control gel). Protein 8 had a pI of ∼10 and a denatured molecular weight of ∼45 kDa. This protein level was increased with ethanol consumption, and further enhanced with betaine supplementation. Proteins 9–11 were downregulated with ethanol consumption with levels not restored by betaine supplementation. These proteins had a pI of 8–8.5 and a denatured molecular weight of ∼28 kDa.

**Fig. 2:**
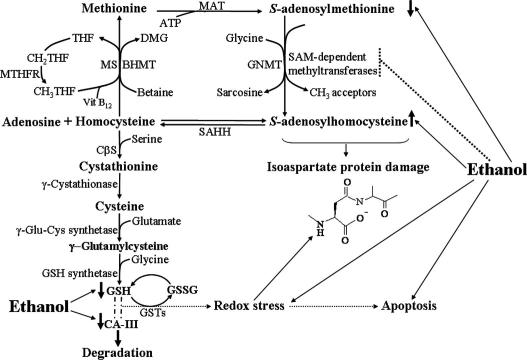
Schematic (simplified) representation of enzymatic and metabolite components of the liver methionine metabolic pathway, and the influence of ethanol consumption. MAT requires ATP for the catalytic production of the methyl donor SAM from methionine. Methylation reactions that use SAM as a methyl donor produce the byproduct SAH. The metabolic ratio of SAM to SAH is modified by GNMT which utilises SAM in the catalytic conversion of glycine to sarcosine. Ethanol consumption results in a disruption in the activities and metabolite levels of methionine metabolic pathway components, and results in a lowering of hepatic SAM levels and an elevation of hepatic SAH levels. Collectively, these metabolite changes compromise methyltransferase activity from both reduced bioavailability of SAM and enzymatic inhibition by SAH. Inhibition of PIMT by this means, results in an elevation of cellular isoaspartate protein damage, which is generally detrimental to protein function. Elevated isoaspartate protein damage may also arise as a result of protein conformational changes induced directly from redox stress. Here we show that betaine supplementation to ethanol-fed rats can trigger the concerted upregulation of BHMT-1, MAT-1, and GNMT, which provide sequential SAM production, and a normalisation of the SAM:SAH ratio. SAH can be hydrolysed by SAH hydrolase (SAHH) resulting in a reversible equilibrium with adenosine and homocysteine. Completion of the cycle in reformation of methionine from homocysteine arises from either vitamin B12 dependent methionine synthase (MS) activity utilising methyl-tetrahydrofolate (THF), or from BHMT activity using betaine and producing dimethylglycine (DMG). Homocysteine can also be catabolised through the transsulfuration pathway catalysed by cystathionine β-synthase (CβS) using serine to form cystathionine. Cystathionine can be further processed via intermediates of cysteine, and then γ-glutamylcysteine, for production of reduced glutathione (GSH). Glutathione is the most prevalent cellular non-protein thiol critical for resisting cellular redox stress by its conjugation to electrophiles thereby forming glutathione disulphide (GSSG). GSSG can subsequently be reduced back to GSH by glutathione reductase. GSTs catalyse the conjugation of endogenous and exogenous electrophiles to glutathione. CA-III is linked to the methionine metabolic pathway via an active formation of two disulphide bonds with glutathione molecules, under conditions of a redox stress insult, with an estimate that CA-III could bind approximately 10% of the total liver glutathione pool if CA-III was completely *S*-glutathiolated [24]. Acute or chronic ethanol consumption results in a reduction in hepatic glutathione levels, and herein we show that ethanol also depletes hepatic CA-III protein levels. Hence we hypothesise that their combined reduction may limit cellular ability to counter subsequent or sustained ethanol-induced redox stress; a component of ethanol-induced apoptotic cell death.

The combination of these three protein separation methods enabled us to characterise 11 prominent liver cytosolic protein changes, and provided information overlap to facilitate verification of protein changes by more than one physical property and separation technique. Thus a ∼45 kDa protein with a pI of ∼10 conforms to the characteristics of protein 1 (by 1D PAGE), protein 4 (by 1D IEF), and protein 8 (by 2D-PAGE). From each of these gel separation techniques, the protein was excised and subjected to MALDI-TOF MS. In all cases this resulted in the identification of the protein as betaine homocysteine methyltransferase-1 (BHMT-1) (see Table 1). BHMT-1 catalyses the conversion of betaine and homocysteine to dimethylglycine and methionine (refer to Fig. 2).

Likewise, both protein 2 (∼35 kDa by 1D PAGE) and protein 6 (pI ∼8.0 by ID IEF) resulted in the identification of this protein as glycine *N*-methyltransferase (GNMT) using MALDI-TOF MS and LC-MS/MS (Table 1). GNMT utilizes SAM as a methyl donor to catalyse the methylation of glycine to form sarcosine (Fig. 2).

Protein 7 which was well resolved by 1D IEF was analysed by MALDI-TOF MS and identified as methionine adenosyltransferase-1 (MAT-1) (Table 1). MAT-1 catalyses the formation of SAM from methionine and adenosine-5′-triphosphate (ATP) (Fig. 2).

Ethanol induced the downregulation of a ∼28 kDa protein (protein 3 by 1D PAGE) with a pI of ∼8.3 (protein 5 by 1D IEF); physical characteristics consistent with protein spots 9–11 from 2D-PAGE. In all cases excision of this protein and analysis by MALDI-TOF MS identified this protein as carbonic anhydrase-III (CA-III, Table 1). CA-III can catalyse the reversible hydration of carbon dioxide to bicarbonate and hydrogen ions, and is a participant in the liver response to redox stress (Fig. 2).

To provide an independent means of validation of this marked ethanol-induced downregulation of CA-III, liver cytosolic proteins from the four feeding regimens were subjected to Western blot analysis using a specific anti-CA-III antibody (Fig. 1D). Densitometric scanning of Western blots revealed the ethanol-induced downregulation of liver cytosolic CA-III to an average of 32 ± 2% of controls.

## Discussion

Models of ALD provide both a molecular insight into ethanol-induced cellular and protein dysfunction, and also afford a viable means to evaluate the usefulness of therapeutic agents aimed at prevention of disease progression. Here we have adopted proteomic approaches to evaluate the most visually prominent protein level changes in rats fed ethanol and betaine supplemented diets. Other minor protein level changes that may arise from these diet regimens were not examined and considered beyond the scope of this study.

From protein profiling of liver cytosolic proteins we were able to characterise upregulation of BHMT-1, MAT-1, and GNMT, with highest increases with the ethanol-fed betaine supplemented rats. This elevation in BHMT-1 protein levels we would expect to drive the remethylation of homocysteine to generate methionine, and this in turn to promote upregulation of MAT-1 and a subsequent increase in SAM levels; levels which are monitored and utilised by GNMT in the methylation of glycine to sarcosine [21] – refer to Fig. 2.

Thus our proteomic study results suggest a hypothesis that betaine supplementation to ethanol-fed rats exerts a concerted influence upon the protein levels of the interconnected methionine metabolic pathway enzymes to drive a normalisation of the SAM to SAH ratio. Noteworthy is that the health benefits of a concerted upregulation of BHMT-1, MAT-1, and GNMT by betaine treatment could counter their collective downregulation in human hepatocellular carcinoma [22].

Our proteomic profiling also fortuitously revealed a dramatic ethanol-induced downregulation of CA-III protein, the levels of which were not restored by betaine treatment. CA-III is a member of the carbonic anhydrase family of zinc metalloenzymes (E.C. 4.2.1.1). Their general attributed function is the reversible catalysis of carbon dioxide hydration to generate bicarbonate and hydrogen ions for maintenance of pH homeostasis, and to aid in the transport of carbon dioxide out of tissues.

However, CA-III is somewhat distinct from the other two cytosolic CA family members (CA-I and CA-II) due to its relatively low carbon dioxide hydratase specific activity, and resistance to inhibition by the sulphonamide, acetazolamide [23]; indicating that CA-III may have other or additional functions. Indeed, CA-III has two reactive sulfhydryl groups (Cys181 and Cys186 that are absent in CA-I or CA-II) that readily form disulphide linkages with glutathione, an active process, termed *S*-(gluta)thiolation, under conditions of redox stress [24–29]. Furthermore, CA-III (but not CA-II) has been shown to protect cells from hydrogen peroxide-induced apoptosis [26]. Taken together, these observations suggest that while CA-III is particularly susceptible to oxidative damage, it may function in hepatocytes and skeletal muscle as an oxyradical scavenger to protect cells from oxidative damage [24–28].

Since ethanol consumption results in cellular redox stress via depletion of glutathione and generation of reactive oxygen species and aldehydes [10–15], we propose that this ethanol-induced downregulation of CA-III levels could further exacerbate any subsequent or sustained ethanol-induced redox stress.

In summary, the health benefits of betaine in part arise through concerted upregulation of methionine metabolic pathway enzymes, but do not extend to maintenance of CA-III levels and its function to resist redox stress.

## Figures and Tables

**Table 1:** Mass spectrometric identification of the most visually prominent protein level changes from rats fed an ethanol diet or an ethanol diet co-supplemented with betaine.

Protein id number	Agent response	Protein identification	Tryptic fragment coverage (%)	Mowse probability score	UniProtKB/Swiss-Prot entry	Protein mass, pI (kDa)
1, 4, 8	Betaine stimulation	Betaine homocysteine methyltransferase-1	53	192	O09171	45, 8.02
2, 6	Betaine stimulation	Glycine *N*-methyltransferase	28	299^∗^	P68369	33, 7.21
7	Betaine stimulation	Methionine adenosyltransferase-1	33	103	P14211	44, 5.61
3, 5, 9–11	Ethanol depletion	Carbonic anhydrase-III	58	178	P14141	29, 6.97

Tryptic fragment coverage from MALDI-TOF MS of the identified proteins is shown, from which a Mowse probability score was generated except GNMT (*) for which the Mowse score was calculated from LC-MS/MS sequence verification of five tryptic peptides. The UniProtKB/Swiss-Prot protein entry number for each of the identified proteins is also included in the table, along with values for the predicted protein mass (in kDa), and theoretical isoelectric point (pI) calculated from protein sequence data.
