# Supplementary material for: Proteomics reveal a concerted upregulation of methionine metabolic pathway enzymes, and downregulation of carbonic anhydrase-III, in betaine supplemented ethanol-fed rats
Source: Biochem Biophys Res Commun. 2009 Apr 17;381(4):523–7. doi: 10.1016/j.bbrc.2009.02.082 (PMC2670967; doi:10.1016/j.bbrc.2009.02.082)
Supplement: Supplementary data [file mmc1.doc]

*Two dimensional polyacrylamide gel electrophoresis (2D-PAGE):-* Six hundred µg of cytosolic liver protein was precipitated with acetone:diethyl ether (2:1 (v/v)) on ice for 1 hour. Precipitated proteins were collected by centrifugation at 5000 rpm (2300 x g) for 3 minutes at room temperature in an Eppendorf 5415R bench centrifuge. Precipitates were washed three times with ether:industrial methylated spirit:water (10:7:2 (v/v/v)), air dried, and then proteins dissolved in a rehydration buffer (9.8 M urea, 2 % (w/v) CHAPS, 0.5 % immobilised pH gradient (IPG) buffer) containing 12 µl/ml of the anti-oxidant destreak reagent (Amersham), at room temperature for 1 hour. Solubilised protein was then actively rehydrated into 7 cm pH 3-10 IPG isoelectric focusing strips for 16 hours at 50 V, and then focussed for 16 hours according to the manufacturer’s guidelines. After focussing, strips were washed in an equilibration buffer of 0.375 M Tris/HCl pH 6.8, 6 M urea, 2 % SDS, 20 % glycerol, containing 2 % (w/v) DTT for 10 minutes, and then similarly washed with the same buffer in which 2.5 % (w/v) iodoacetamide replaced the DTT. After this reduction and alkylation, strips were equilibrated in MES running buffer before layering onto the top of Novex 4-12 % Bis-Tris Zoom gels. Strips were fixed with 1 % (w/v) molten agarose, and then proteins resolved under conditions described for 1D SDS-PAGE. Resolved proteins were stained with colloidal Coomassie Brilliant Blue overnight according to the manufacturer’s guidelines. Gels were destained with water, and then photographed using a Fugi E900 digital camera.

*Matrix assisted laser-desorption ionisation-time of flight (MALDI-TOF) mass spectrometry:-* Protein bands or spots stained with Coomassie from either, 1D PAGE, 1D IEF, or 2D-PAGE gels were excised from the gels and transferred into a 96-well plate using an automated MassPrep robotic system (Proteome Works, Bio-Rad, UK). Gel pieces were destained, reduced and then alkylated with DTT and iodoacetamide respectively, before digestion *in situ* with trypsin. Tryptic fragments were desalted by binding and then elution from a C18 Zip-tip (Millipore), and then mixed with α-cyano-4-hydroxycinnamic acid (Sigma C-2020) matrix solution. Peptides then underwent laser desorption and MALDI-TOF mass spectrometry analysis using a Micromass MALDI (Waters, UK). Intact singularly charged peptides were identified and their mass used in a search algorithm (MASCOT peptide mass fingerprint) to screen protein databases such as Swiss-Prot for tryptic peptide matches. A Mowse probability score of greater than 66 was considered a significant match (*P* < 0.05) of tryptic peptides to those present within protein databases, to enable protein identification. MALDI-TOF MS analyses were performed for each protein band or spot from multiple replicate gels, for which the highest tryptic coverage from a single MALDI-TOF analysis is shown in Table 1.

*Liquid chromatography mass spectrometry/mass spectrometry (LC MS/MS):-* Stained protein spots were excised, destained, reduced, alkylated, and tryptic peptides produced according to the above described MALDI-TOF method. Extracted tryptic peptides were desalted and then run on a Waters QTOF2 hybrid quadrupole mass spectrometer incorporating an integrated capillary LC system. Tryptic fragments were initially desalted on a C18 pre-column and then loaded onto and eluted from a 100 mm x 0.75 mm internal diameter C18 analytical capillary column. The LC system solvent flow rate was 200 nl/min, with a gradient of 60 minutes used for column equilibration and peptide elution. Eluted peptides were submitted into the mass spectrometer directly via a nanosprayer attached to the outflow from the LC system, and operating at 3 kV. A separate nanosprayer also submitted a reference solution containing a peptide of known mass into the mass spectrometer. This ion source was sampled at regular intervals throughout the sample run to assist with maintaining accurate mass measurements of the ionized peptides from the analyte spray. Data-dependent switching was incorporated so that whenever a peptide with an associated charge of 2+ or 3+ was detected above a preset threshold signal, the mass spectrometer would then automatically switch to MS/MS mode to generate fragmentation data from the detected peptide. A preset range of collision voltages was used to fragment each peptide as efficiently as possible. For co-eluting peptides, software was enabled that allowed fragmentation data from each peptide to be collected simultaneously, but individually stored for each peptide. Peptide MS/MS raw data files were analysed using MassLynx 4.0 (incorporating BioLynx) and ProteinLynx Globalserver 2 (PLGS2) (Waters) in order to assess the identities of proteins present in the digest. The peak list file generated from PLGS2 analysis was also used in alternative search engines accepting this format of data file including MASCOT. Fragmentation data was also analysed manually and *de novo* sequencing performed on selected peptides.
